# Identification and Validation of Common Reference Genes for Normalization of Esophageal Squamous Cell Carcinoma Gene Expression Profiles

**DOI:** 10.1155/2022/9125242

**Published:** 2022-11-23

**Authors:** Jia Xu, Ming Yang, Ai-zhong Shao, Hui-wen Pan, Yi-xuan Fan, Ke-ping Chen

**Affiliations:** ^1^School of Life Sciences, Jiangsu University, Zhenjiang, China; ^2^Department of General Surgery, Fifth People's Hospital of Huaian City, Huaian, China; ^3^School of Food and Biological Engineering, Jiangsu University, Zhenjiang, China; ^4^Department of Cardiothorac Surgery, Affiliated People's Hospital of Jiangsu University, Zhenjiang, China; ^5^Department of Cardiothoracic Surgery, Affiliated People's Hospital of Jiangsu University, Zhenjiang, Jiangsu Province, China

## Abstract

Esophageal squamous cell carcinoma (ESCC) is one of the subtypes of esophageal cancer with Chinese characteristics, and its five-year survival rate is less than 20%. Early diagnosis is beneficial to improving the survival rate of ESCC significantly. Quantitative Real-Time Polymerase Chain Reaction is a high-throughput technique that can quantify tumor-related genes for early diagnosis. Its accuracy largely depends on the stability of the reference gene. There is no systematic scientific basis to demonstrate which reference gene expression is stable in ESCC and no consensus on the selection of internal reference. Therefore, this research used four software programs (*The comparative delta-Ct method*, *GeNorm*, *NormFinder*, *and BestKeeper*) to evaluate the expression stability of eight candidate reference genes commonly used in other tumor tissues and generated a comprehensive analysis by RefFinder. Randomly selected transcriptome sequencing analysis confirmed the SPP1 gene is closely related to ESCC. It was found that the expression trend of SPP1 obtained by RPS18 and PPIA as internal reference genes were the same as that of sequencing. The results show that RPS18 and PPIA are stable reference genes, and PPIA + RPS18 are a suitable reference gene combination. This is a reference gene report that combines transcriptome sequencing analysis and only focuses on ESCC, which makes the quantification more precise, systematic, and standardized, and promotes gene regulation research and the early diagnosis of ESCC in the future.

## 1. Introduction

Esophageal cancer is one of the malignant digestive system tumors with high lethality. Esophageal squamous cell carcinoma (ESCC) and esophageal adenocarcinoma (EACH) are the two most common histological types [1–3]. ESCC is a malignant tumor with hidden early symptoms and Chinese characteristics. The 5-year survival rate of early esophageal cancer can exceed 95%. But the overall 5-year survival rate is less than 20%, which seriously reduces the happiness of life of the people [4]. Precision medicine is a new medical concept and medical model based on the idea and technology of precision screening, diagnosis, and treatment. Precise screening is the premise and effective means of early diagnosis, which brings new hope for improving the survival rate [5, 6].To confirm the existence of cancer, real-time quantitative Polymerase Chain Reaction from suspected tissues to confirm the abnormal expression of tumor-related genes is a very productive screening method [7].

Quantitative Real-Time Polymerase Chain Reaction (qRT-PCR) can monitor the expression of target genes in real time, commonly used in the field of molecular biology currently. It has unique advantages such as high sensitivity, simple operation, and reliable results [8, 9]. qRT-PCR is often used in pathogen detection, signal transduction, and the effect of disease on gene expression, which solves many focal issues in disease pathogenesis and cancer treatment in the field of medicine [10–12]. The accuracy of qRT-PCR is affected by varieties of factors [13, 14]. Normalization of the gene of interest with appropriate reference gene data is critical to minimize bias associated with qRT-PCR results. Accordingly, selecting a reference gene whose expression level does not vary significantly with sample type, developmental stage, or experimental treatment is essential. Housekeeping genes meet the selection criteria of general reference genes in principle, but so far, do not have a housekeeping gene that can be applied to all conditions [15].

In tumor-related gene expression and transcription analysis, the most commonly used reference genes can no longer meet the requirements of accurate quantification and need to be determined by screening and evaluation [16, 17]. Used keywords to query the PubMed database and found reference genes commonly used in the molecular biology research of ESCC were *actin* beta (ACTB) [18, 19]and glyceraldehyde-3-phosphate dehydrogenase (GAPDH) [20, 21], such as “esophageal squamous cell carcinoma,” “real-time,” and “PCR.” ACTB encodes an actin gene that is widely present in nonmuscle cells and participates in the formation of the cytoskeleton and cell movement [22]. GAPDH is a key enzyme in the process of gluconeogenesis and glycolysis in a variety of organisms [23]. A study by the Cancer Research Institute found that ACTB is the best reference gene for quantification in some cancer tissues [24]. Conversely, studies have also found that ACTB is upregulated in most tumor cells and tissues [25]. This site proves that no internal reference gene can be completely suitable for quantification under all experimental conditions. Relevant literature does not support why ACTB and GAPDH are used and whether they are accurate and representative of ESCC. With the development of bioinformatics, the frequency of use of qPCR is getting higher. Some institutions have begun to pay attention to the excavation of the stability of internal reference genes (http://icg.big.ac.cn) [26]. But ESCC tissues and cell lines have not been included in the existing database, which means that there is no consensus, systematic, and scientific evidence on which reference gene should be selected for the gene research of ESCC. Precision medicine is more focused on the guidance of precise concepts and technologies. The tumor is so complex that the expression profiles of diverse experimental materials with mechanopathological features will be different [27]. For example, blood differs from tissues [28]; animals and human being will also be different [29]; and as the tumor grows, the IFP values and some gene expression level also increase [30]. Therefore, the reference genes should be accurately selected and refined according to the experimental materials and research purposes [15].

Cancer researchers generally compare tissue gene expression for subsequent in vitro studies. Therefore, this research focused on the stability of reference genes between ESCC tissues and paired normal esophageal tissues. Firstly, find eight commonly used reference genes in tumor tissue as candidate genes in the PubMed database. In addition to ACTB and GAPDH prevalently mentioned, there are ribosomal protein S18 (RPS18) [31, 32], beta-2-microglobulin (B2M) [33, 34], hypoxanthine phosphoribosyl transferase 1 (HPRT1) [35, 36], glucuronidase beta (GUSB) [37–40], peptidylprolyl isomerase A (PPIA) [41, 42], and phosphoglycerate kinase 1 (PGK1) [43, 44]. Secondly, design and specificity analysis experiments were carried out to verify primer, qRT-PCR was performed, and the experimental results were evaluated by five kinds of expression stability software, such as *GeNorm*, *NormFinder*, and so on. Finally, transcriptome sequencing was performed for differential expression analysis, the target genes were screened, and patient samples were randomly selected to verify the stability results of the obtained internal reference gene.

## 2. Material and Methods

### 2.1. Human Tissues

Two hundred and ten matched pairs of normal and esophageal tumor tissues were obtained by endoscopic resection during the examination of the patients who gave informed consent (the tumor margin ≥5 cm and the farthest distance ≤7 cm to reduce errors and ensure balanced comparability). And, clinical data of patients were collected for statistical analysis. All procedures followed the protocol approved by the Medical Ethics Committee of Jiangsu University and followed the Helsinki Principles and relevant Chinese policies and regulations. Rinse the specimens with 0.9% normal saline before sampling, put them in a cryopreservation tube with RNA-EZ reagents preservation solution (Sangon Biotech, China) added immediately after sampling, and store them with liquid nitrogen, and store them in a -80°C refrigerator for later use.

### 2.2. Quantitative Real-Time Polymerase Chain Reaction (qRT-PCR)

Accomplished RNA extraction, cDNA synthesis, reference gene selection (Supplementary 1, Table S1), and primer design, we adopted primers of amplicon length below 200 bp, except for GUSB, to maintain consistency in amplification efficiency [45] (Table S2). qRT-PCR was performed using 2 *μ*l of tenfold diluted cDNA, 0.4 *μ*l of 10 *μ*M of each primer, and 5 *μ*l of 2 × AceQ Universal SYBR qPCR Master Mix in the final volume of 10 *μ*l. The PCR cycle conditions were set as follows: preincubation for 5 minutes at 95°C followed by 40 cycles, with each cycle including 10 seconds at 95°C and 30 seconds at 60°C. And, the melting curve program is 15 seconds at 95°C, 60 seconds at 60°C, and 15 seconds at 95°C; relative quantifications were performed by Q3 (Thermo Fisher Scientific, UK). The gene expression levels for each sample were determined based on three replicates following the MIQE (Minimum Information for Publication of Quantitative Real-Time PCR Experiments) guidelines.

### 2.3. qRT-PCR Experimental Data Analysis

The cycle threshold (Ct) value was recorded for each qRT PCR gene expression analysis. Statistical analyses were performed with GraphPad Prism 8 (GraphPad Software, La Jolla, CA, USA). Scatter plots were drawn to visualize the reference gene expression levels and variations. The comparative *delta-Ct* method [46, 47], *GeNorm*[47–49], *NormFinder*[50, 51], *BestKeeper*[52–55], and *RefFinder*[56] algorithms were used to assess the expression stability of selected genes. The specific methods of the five algorithms are shown in Supplementary 1.

### 2.4. Transcriptome Sequencing and Assembly

RNA-seq transcriptome strand library was prepared following TruSeqTM Stranded Total RNA Library Prep Kit from Illumina (San Diego, CA) using 5 *μ*g of total RNA. Libraries were selected for cDNA target fragments of 200–300 bp on 2% Low Range Ultra Agarose followed by PCR amplified using Phusion DNA polymerase (NEB) for 15 PCR cycles. After quantified by TBS380, the paired-end RNA-seq sequencing library was sequenced with the Illumina HiSeq X ten. The raw paired-end reads were trimmed and quality controlled by SeqPrep (https://github.com/jstjohn/SeqPrep) and Sickle (https://github.com/najoshi/sickle) with default parameters. Then, clean reads were separately aligned to the reference genome with orientation mode (GRCh38.p13,http://asia.ensembl.org/Homo_sapiens/Info/Index) using HIASAT (https://ccb.jhu.edu/software/hisat2/index.shtml) software. The mapped reads of each sample were assembled by StringTie (https://ccb.jhu.edu/software/stringtie/index.shtml?t=example) in a reference-based approach.

### 2.5. Quantification of Gene Expression Levels and Validation of Reference Genes

To confirm the reliability of reference genes, transcriptome sequencing results were quantified. The expression level of each transcript was calculated according to the Transcripts Per Million reads (TPM) method based on the length of the gene and the number of reads mapped to the gene. RSEM (http://deweylab.biostat.wisc.edu/rsem/) was used to quantify gene abundances. The fold difference (FC) > 2, *p* < 0.05 was used as the standard to screen for expression difference analysis, and then the protein-protein interaction was performed from the differential genes to screen out the expression of important genes with significantly increased expression and more related to the pathogenesis of ESCC. Then, normalize the selected gene of a target with the two most stable and least stable reference genes[57, 58]. The qRT- PCR amplification conditions were as described, and the gene expression was calculated using the experimental group/control group = 2^−△△Ct^. Calculate the relative expression levels of each reference gene and target gene, respectively, take the geometric mean of the two as the standardization result of double reference genes[59, 60], and use SPSS18 software for statistical analysis.

## 3. Result

### 3.1. Patient Clinical Data Statistics

**Figure 1:**
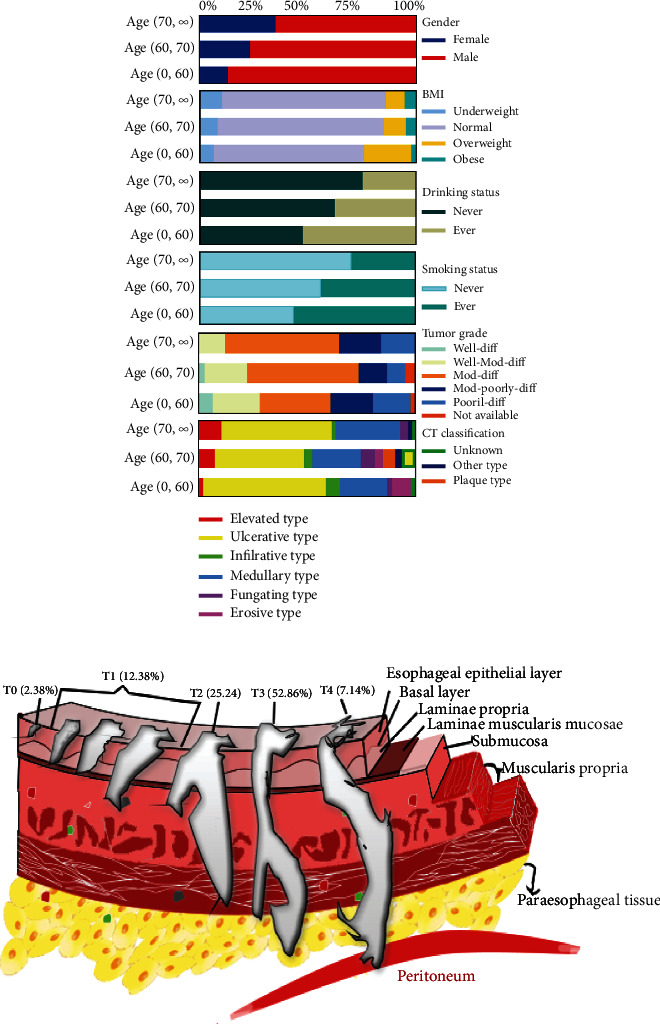
Information statistics. Note: Stage classification follows the TNM classification system by International Union Against Cancer (UICC) [63].

A total of 210 patients with ESCC were included in this research, with a wide age range and obvious individual differences. ESCC is more common in middle-aged and older people, with a high incidence in 61-70 years old [61, 62]. Referring to the age segmentation method in China, the patients were divided into young and middle-aged groups (less than or equal to 60 years old, YM), young and elderly groups (age between 61 and 70 years old, YE), and elderly groups (age over 70 years old, E). The differences in gender, BMI, smoking, drinking, differentiation, and pathological types of patients were compared among the three groups (Figure 1). The results showed that 46 patients (21.9%) were in group YM, 107 patients (50.9%) were in group YE, and 57 patients (27.2%) were in group E. And when all patients in the T stage of the staging were counted, 5 cases (2.38%) were in the T0 stage, 26 cases (12.38%) in the T1 stage, 53 cases (25.24%) in the T2 stage, 111 cases (52.86%) in the T3 stage, and 15 cases (7.14%) in the T4 stage (Figure 1(b)). As indicated in Figure 1(a), there were differences in the clinical characteristics of the three groups of patients. There were more males than females, fewer nonsmokers, and fewer drinkers than smokers and drinkers. The diagnosis of esophageal cancer is mainly in the middle and later stages, and the ulcerative type and the medullary type account for a large proportion of the pathological classification. As a result, the samples included in this experiment can provide better sample support for the stability of subsequent reference genes.

### 3.2. Expression Level of Candidate Reference Genes

**Figure 2:**
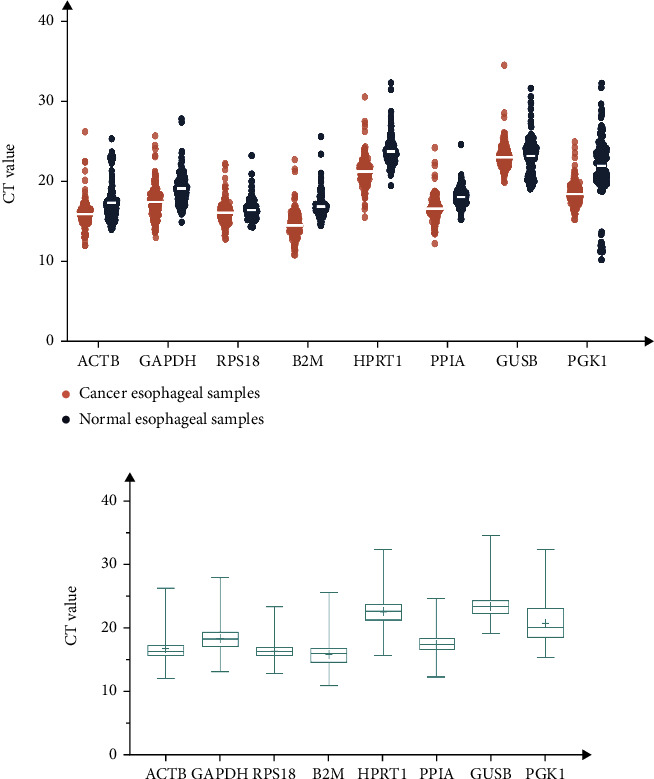
Expression levels for eight candidate reference genes detected by RT-qPCR. (a) Individual CT distribution; (b) all esophageal tissue samples. Note: The higher the Ct value, the lower the expression level. The box indicates the 25th and 75th percentiles, with the line across the box representing the median. The plus sign represents the mean value.

RNA quality detection and primer-specific identification can meet gene stability verification requirements (Figure S1, Table S1). The Ct value can characterize the mRNA transcription level. qRT-PCR was performed on RNA samples extracted from all individuals, and the results were plotted as a scatter plot (Figure 2). The average Ct values of cancer and normal tissues were between 14.72 and 23.78, implying a large difference in gene expression. ACTB and RPS18 displayed a high abundance in cancer and normal tissues. In contrast, GUSB observed the lowest expression level (Figure 2(a)). Further normality evaluation of the aforementioned results, by the *k*-*s* test and D'Agostino and Pearson's omnibus normality test, demonstrated that the reference gene does not appear to have a Gaussian normal distribution in both cancer tissue and normal tissue. To compare nonnormally distributed paired groups, we performed nonparametric Wilcoxon text except for GUSB (*p* = 0.3189). The remaining seven candidate reference genes were significantly reduced from normal esophagus to tumor esophagus (*p* < 0.05).

Box plots were used to present the distribution of the Ct values for all samples (Figure 2(b)). Five genes (ATCB, GAPDH, B2M, RPS18, and PPIA) were highly expressed, with mean Ct values ranging from 15.5 to 20. At the same time, three genes (GUSB, PGK1, and HPRT1) were moderately expressed with Ct values between 20 and 24. B2M (average Ct is 15.82) and GUSB (average Ct is 23.35) had the highest and lowest expression levels. Simultaneously, PGK1 and RPS18 detected the most and the least variable expression, respectively, indicating the stability of expression levels. PGK1 was the most erratically expressed gene, while RPS18 was the most stably expressed gene.

### 3.3. Expression Stability of Candidate Reference Genes

To identify the most stable reference genes, this research used five algorithms to analyze qRT-PCR expression data, the comparative *delta-Ct* method, *GeNorm*, *NormFinder*, *BestKeeper*, and *RefFinder*. The different algorithms analysis results are as follows.

#### 3.3.1. The Comparative *Delta-Ct* Method Analysis Result

**Figure 3:**
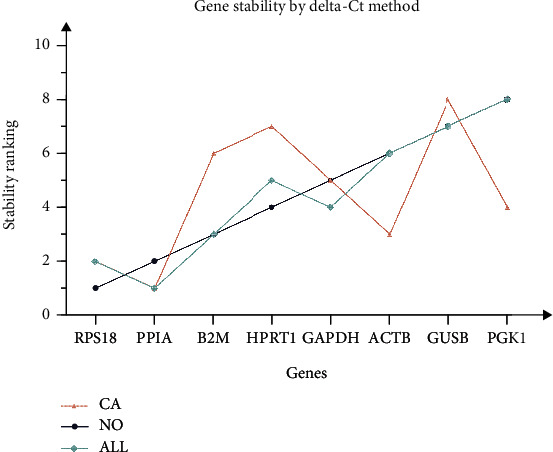
Stability ranking of the expression stability of the candidate internal reference genes by *Delta-Ct* method.Note: NO: normal esophageal tissues; ALL: all esophageal tissues; CA: esophageal cancer tissues.

Here, we employed a comparative *delta-Ct* method by comparing the relative expression of pair of genes within each sample to identify useful housekeeping genes confidently. The overall ranking is shown in Figure 3. PPIA had the most stable expression in esophageal cancer tissues (CA) and all esophageal tissues (ALL); RPS18 had the most stable expression in normal esophageal tissues (NO). Meanwhile, the expression of PGK1 was the most unstable in group NO and ALL; and GUSB in CA.

#### 3.3.2. *GeNorm* Analysis Result

**Figure 4:**
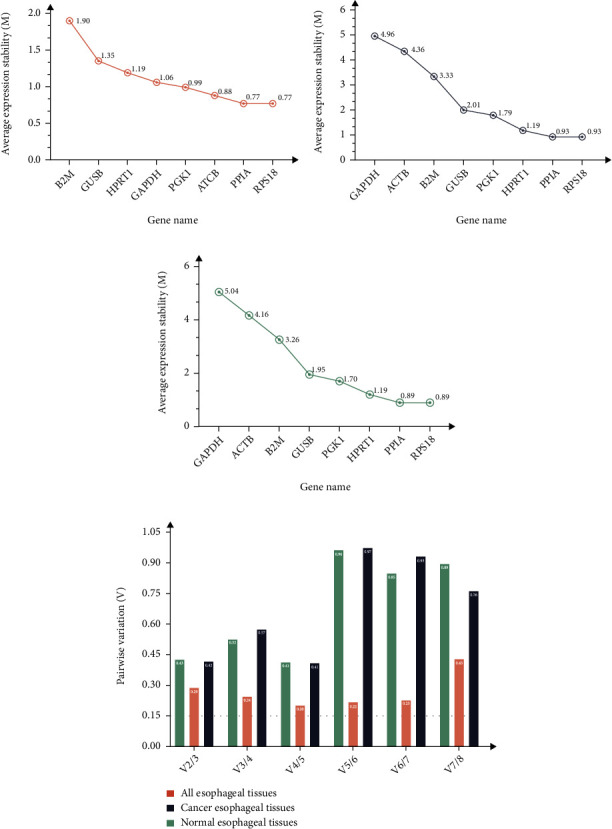
Average expression stability and pair-wise variation analysis of eight candidate reference genes by *GeNorm* analyses. Expression stability was plotted in CA (a), NO (b), and ALL (c); pair-wise variation analysis (d). Note: The least stable reference gene (higher *M* value) is on the left, and the most stable combination (lower *M* value) is on the right of the plot. Most stable reference genes were deduced by the stepwise exclusion of the least stable genes. All the pair-wise variations were under the default limit of 0.15.

The *M* value refers to the average variation of a gene relative to other genes, so we set the threshold for gene stability as *M* < 1.5 to assess the stability of the reference gene. The lower the *M*-value, the higher the candidate gene stability. The results showed that, with the exception of B2M, the remaining seven candidate genes in esophageal cancer tumor tissue all met the stable gene (M1.5) standard (Figures 4(a)–4(c)). PPIA and RPS18 had the highest stability; in paired normal esophageal tissue and all tissues, only the M of PPIA, RPS18, and HPRT1 was less than 1.5, indicating that the expression of these three genes was stable. Combining the three conditions, *GeNorm* analysis concluded that PPIA and RPS18 were the most stably expressed genes, which was similar to the *Delta-Ct* analysis results, and GAPDH was the most unstable gene, contrary to the *Delta-Ct* analysis results. As a consequence, there were differences in the results obtained by the two analytical methods.

To determine whether these eight genes have the optimal number of internal reference, genes in ESCC, N_Fn_, and N_Fn+1_ were used in the *GeNorm* program to calculate Vn/Vn + 1 values for different candidate gene pairs and to determine whether increasing the number of internal reference genes can improve the stability of the normalization factor simultaneously. All data were imported into the *GeNorm* program to obtain Vn/Vn + 1, dig out that Vn/Vn + 1 exceeded 0.15, and the optimal number of reference genes was not determined for the three treatments (Figure 4(d)).

#### 3.3.3. *NormFinder* Analysis Result

We also applied the same dataset using the *NormFinder* package by R Studio and calculated stability values (SV). *NormFinder* can calculate the SV of each reference gene based on the intragroup and intergroup variation [64]. Lower SV corresponds to higher stability, stability ranking as shown in Table 1. For group CA and NO, PPIA and RPS18 were the reference genes with the most stable expression. While GUSB was the most unstable reference gene for all samples, GAPDH and PGK1 were the most stable and least stable reference genes.

**Figure 5:**
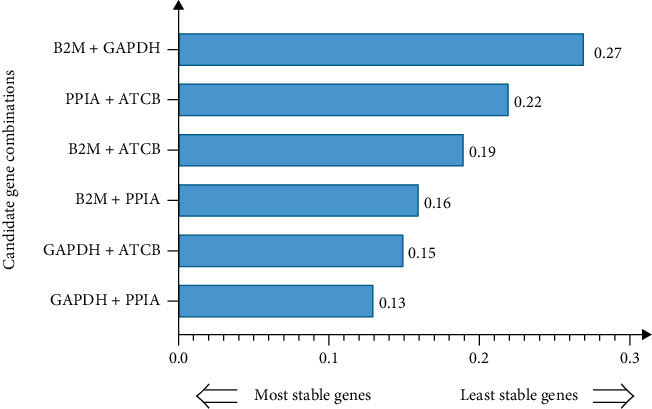
Evaluation of the expression stability of the candidate internal reference genes combinations by *NormFinder.*

For grouped sample comparison (such as gene comparison between ESCC tissue and normal tissue), *NormFinder* screening recommends the use of a reference combination to increase the reliability of the reference gene and reduce intra- and intergroup variability. Since the SV of the first four candidate genes was very close (GAPDH, PPIA, ATCB, and B2M), the aforementioned four genes were paired to evaluate the stability of all samples. Taking SV < 0.3 as the screening criterion (Figure 5), it illustrated that the combination of top2 genes (GAPDH and PPIA) manifested smaller changes between the control group and the patient group, and the expression stability of the gene was the highest (SV = 0.13). It can be used for standardization of subsequent qRT-PCR analysis.

Similarly, the rankings generated by NormFinder were very similar to the Delta-Ct analysis for individual esophageal cancer tissues and normal tissues. However, all samples were inconsistent. For example, RPS18 was one of the most stable genes in the Delta-Ct and GeNorm analysis but the third stable gene in the NormFinder analysis. The top2 gene combination obtained by NormFinder also contradicted the result that the number of reference genes of GeNorm optimal number was not determined. It was worth considering in the subsequent verification of the expression stability of candidate internal reference genes.

#### 3.3.4. *BestKeeper* Analysis Result

*BestKeeper* software calculates stability based on standard deviation (SD), Pearson's correlation coefficient (*r*), and the coefficient of variation (CV) of gene Ct. The smaller the value of std dev [±CP] of the gene, the more stable the expression [65]. In this study, the expression stability ranking was obtained by the size of std dev [±CP] (Table 2). The ranking demonstrated that RPS18 was the most stably expressed gene in group ALL; PPIA was in a single group of normal and tumor tissue with std dev [±CP] less than 1.0. GAPDH was the most erratically expressed gene in group CA but ranked not as low as PGK1 in normal tissue and all tissue samples. From the data, the stability level of candidate reference genes generated by *BestKeeper* was similar to that of *NormFinder*. A few genes were different. For example, in group ALL, GAPDH was identified as the most stable intrinsic gene in the *NormFinder* analysis, while it ranked third from the bottom in the *BestKeeper* analysis.

#### 3.3.5. *RefFinder* Analysis Result

**Figure 6:**
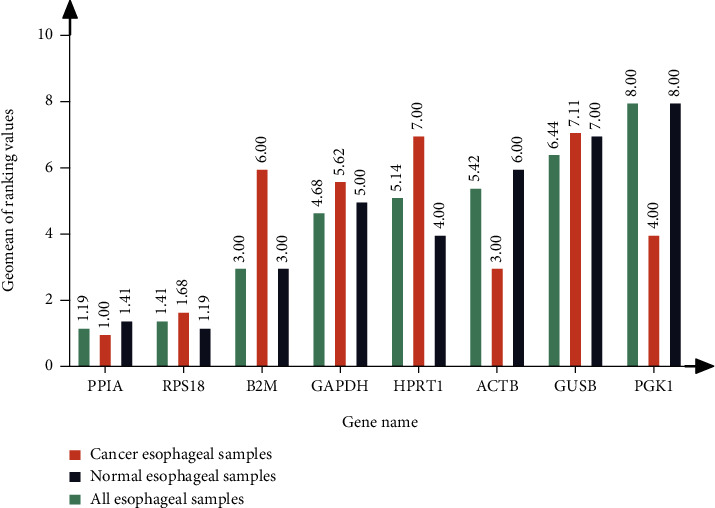
Comprehensive ranking of stability.

It can be seen that the different algorithms and judgment standards between the software and the expression stability coefficients of the same reference gene obtained by different software were inconsistent. Finally, the eight candidate reference genes were comprehensively ranked using the stability comprehensive analysis software *RefFinder*[65] (Figure 6). The results illuminated that the two reference genes, PPIA and RPS18, were the most stably expressed under various conditions, and GUSB was the most erratically expressed gene. At the same time, PGK1 also performed instability in esophageal cancer samples and all esophageal samples, and HPRT1 was also unstable in normal esophageal samples. The use of multiple internal references can effectively reduce the test error. Under the conditions of this experiment, the combinations of two suitable internal reference genes of cancer esophageal tissues, normal esophageal tissues, and all samples were PPIA and RPS18.

## 4. Sequencing Data Quality Control and Differentially Expressed Gene Analysis

**Figure 7:**
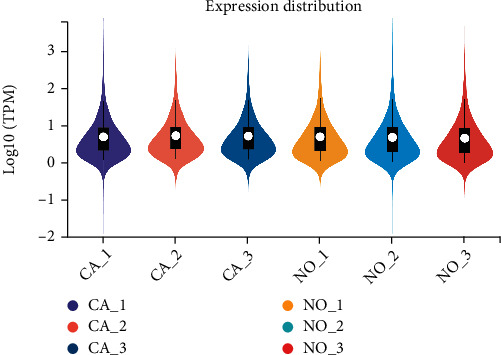
Transcriptional expression profiles of sequenced samples.Note: Three biological replicates.

The raw data obtained by sequencing was controlled by *fastp*, and the expression distribution of the transcriptome in each sample was shown in Figure 7. Both Q20 and Q30 were above 96.09%, and the GC content was normal. Sequenced alignment of clean data and *Homo_sapiens* genome (GRCh38.p13, http://asia.ensembl.org/Homo_sapiens/Info/Index). The alignment efficiency ranged from 91.96% to 96.05%, and the sequencing quality was high, which could be used for follow-up analysis.

**Figure 8:**
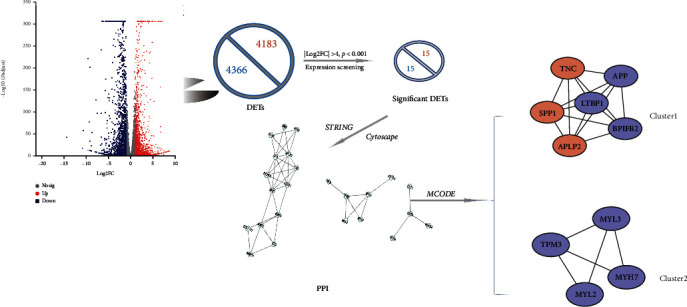
Differential gene protein-protein interaction and screening of target genes.Note: Red represents transcripts with increased expression in tumor tissue; blue represents transcripts with reduced expression in tumor tissue; DETs: differentially expressed transcripts.

FC > 2, *p* adjust <0.05 as the screening criteria, differential analysis of transcripts in CA and NO samples using DESeq2, and a total of 8549 differential mRNAs were obtained (Figure 8). The screening conditions are continuously optimized to identify the significant differentially expressed genes related to ESCC. FC was adjusted to be greater than 16 times, that is, |Log2FC| > 4, *p* adjust <0.001, and the expression level was also considered for screening. Then the interaction relationship of these genes was extracted from the *String*; *Cytoscape* was used to construct protein-protein interaction (PPI), and MCODE plug-in was used to select the key subnetworks and to get the map (Figure 8). It can be seen that many genes were associated with the upregulated gene SPP1 (secreted phosphor protein 1, SPP1), which was in the center of the cluster, indicating that the greater probability was related to the occurrence or diagnosis of ESCC. This research consequently chose the central gene SPP1 as the follow-up verified target gene (Table 3).

## 5. Target Gene Expression Profiles Are Influenced by Reference Genes Employed for Normalization

**Figure 9:**
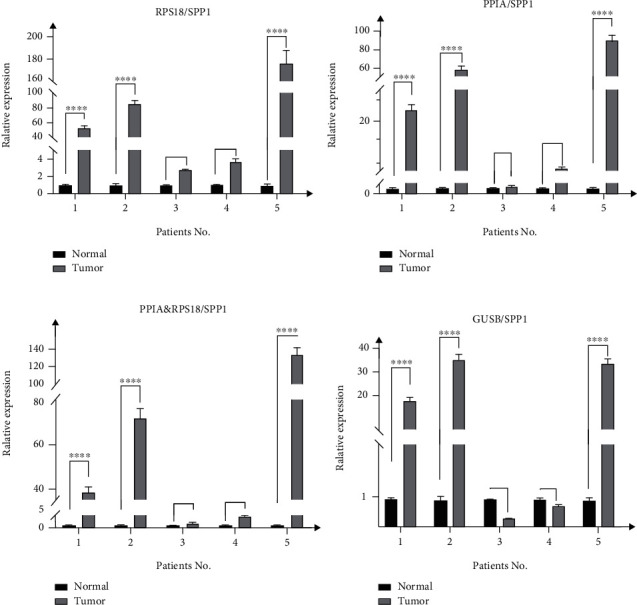
Relative expression of SPP1 normalized by reference genes in ESCC and paired normal tissues.

To evaluate reference genes in practical situations, this study randomly selected ESCC tissues and paired normal esophageal tissues from several groups of patients for in vitro studies. We standardized the relative expression (RE) of the target gene SPP1 in tumor tissue and normal tissue by combining a single reference gene and two combined genes. The RE results are shown in Figure 9. From the results of transcriptome sequencing (Table 3) and other research [66, 67], SPP1 is a secreted protein that promotes tumor proliferation and is higher in tumor tissue than in normal tissue. Consequently, there should be an upward trend in the expression from normal tissue to cancer tissue. When a stably expressed single internal reference is used for normalization, RPS18 and PPIA, the high and low patterns of RE between normal and tumor were similar (Figures 9(a) and 9(b)), and the trends were the same as the sequencing results. On the other hand, the RE of GUSB represented a different pattern from the high-ranked single reference genes (PPIA and RPS18) in some patients, and the trend was opposite to the sequencing results (Figure 9(d)). The relative expression normalized by the geometric mean of the internal control combination (PPIA and RPS18) displayed a similar pattern to the single internal controls (PPIA or RPS18), except that RE was in between (Figure 9(c)). From these results, it can be seen that under the standardization of different internal reference genes, the relative expression of SPP1 will be different in different patients, which implies that the expression of SPP1 does vary. The number difference in the expression level of SPP1 between the two groups depends on the individual differences of patients. We speculate that the different degrees of cancer differentiation and pathological types of patients may lead to different expression levels [68]. This is a problem that several computational models in the field of cancer research also mentioned and assumed [30].In general, these experimental results not only reveal that the stability ranking results of internal reference genes obtained by the evaluation software are reliable, RPS18 and PPIA can be used for the identification of target genes in ESCC tissues, but also reveals the possibility of a new biomarker for esophageal squamous cell carcinoma, which can analyze tumor staging, reclassification, treatment response evaluation, and even predict the risk of certain diseases by measuring SPP1 concentration in tissues.

## 6. Discussion

Esophageal squamous cell carcinoma is a malignant tumor with Chinese characteristics. Epidemiological characteristics are often related to factors such as diet (drinking, smoking, chewing betel nut, and drinking very hot beverages), BMI, and other factors [69–71]. After more than 70 years of development, China has made remarkable achievements in preventing and treating esophageal cancer and is now at the top level in the world. The early symptoms of this cancer are very subtle or lacking, so it is often diagnosed after the disease has deteriorated significantly, which makes the early diagnosis rate only 1.43%. With the development of bioinformatics, transcriptomic studies have found that some genes involved in tumorigenesis and metastasis are differentially expressed in tumor and normal tissues [72–75]. This opens the possibility for early detection of esophageal squamous cell carcinoma. qRT-PCR analysis has been widely used to quantify target gene transcription levels. And it has proven to be a robust and specific method for characterizing gene function and confirming the identity of candidate genes in esophageal cancer pathogenesis [76–78]. This technique can detect weak signals from very small biopsy samples when patients are in the early stages of cancer. Paradoxically, the accuracy of qRT-PCR gene expression analysis mainly depends on the selection of appropriate internal controls (i.e., reference genes). In the absence of appropriate reference genes, the data obtained may be misleading. Reference genes were initially selected based on the function of housekeeping genes.

After the twentieth century, with the development of genomics technology, some nonhousekeeping genes could also become internal reference genes under certain experimental conditions. Prior to this study, no valid reference genes for ESCC or esophageal tissue had been identified. ACTB and GAPDH are now most frequently used [79–83]. But even so, it does not premeditate whether they are expressed inconsistently in different experimental settings and clinical conditions. Some studies have found that ACTB expression is not stable in tumors, and some researchers have proposed that there is no completely constant reference gene in various tissues and cells [25, 84, 85]. For future research on the expression of candidate genes in the pathogenesis of ESCC and to ensure the accuracy of qRT-PCR gene expression analysis, we tested, in addition to ACTB and GAPDH, six other commonly used genes that have been evaluated as stable reference genes in other human cancer studies for the purpose of providing stable reference genes in ESCC. Five algorithms, including the comparative *delta-Ct* method, *BestKeeper*, *GeNorm*, *Normalfinder*, and *RefFinder*, were used to analyze its expression stability in 210 pairs of esophageal squamous cell carcinoma tissues and normal esophageal tissues with complex clinical features, and the analysis results were verified by sequencing.

We ensure the accuracy and objectivity of the research results from various aspects so that the results can be widely used. Firstly, the samples used in this experiment were large in number and complex in characteristics (Figure 1). The samples included a wide range of age groups (high-incidence and nonhigh-incidence ranges), multiple sex (male and female), broad-spectrum BMI (underweight to obese), differentiated eating habits (whether smoking or drinking), different degrees of differentiation (mild to severe), various of pathological types, and T stages. Thus, the number of samples meets the experimental requirements, and the pathological characteristics meet one of the conditions for the general and stable expression of the internal reference gene. Secondly, to ensure the accuracy, relevance, and correctness of qRT-PCR, this experiment not only strictly abides by MIQE for each gene and each sample in triplicate but also tries to eliminate or reduce the influence by the number and quality of templates, RNA extraction, cDNA synthesis efficiency, and qPCR amplification (Figure S1, Tables S1 and S2) [86, 87]. For example, the number of samples was much larger than the number of genes. As a consequence, we used sample maximization on the RT-qPCR layout to eliminate errors in each quantification. We used sterile, fast, and correct extraction methods to get rid of the genome when extracting RNA (it eliminates the effect of competitive binding of primers [88, 89]). The obtained 420 individual RNA nucleic acid gel bands were clear and in the correct position. Most of the A260/A280 was distributed around 2.0 (Figures S1(a) and S1(c)), indicating that the RNA was pure and free of contamination. Then, the same amount of RNA was used for reverse transcription in the same batch to ensure stable synthesis efficiency. Maintaining a similar amplification efficiency for the reference gene and the target gene is necessary. At the same time, the amplification efficiency was closely related to the primer length. Shorter primers generally have higher efficiency. It was found that the shorter amplicon (70-250 bp) in RT-qPCR was “independent” of RNA quality, and the amplification efficiency of primers around 200 bp was consistent in the previous research [45, 90, 91]. Undoubtedly, this experiment not only controls the length of primers within 200 bp but also verifies whether the amplification efficiency of these eight pairs of primers is similar. The experimental results illustrate that the eight pairs of primers have good specificity and excellent amplification efficiency (E: 95.8% ~103.2%) (Table S2).

Furthermore, we used five existing internal reference gene stability assessment tools to evaluate gene stability in all aspects and from multiple perspectives. The four algorithms (except *RefFinder*) were based on different models and assumptions. Each algorithm produced different results for the expression data of the same gene [92, 93]. Then, RefFinder was used to calculate the recommended comprehensive ranking based on the results of the four algorithms as mentioned. The obtained internal reference genes were more stable and suitable for gene research in ESCC (Figures 2–6, Tables 1 and 2). In order to increase the credibility of the obtained reference genes, we performed transcriptome sequencing of the two mixed tissues to obtain the expression profiles of esophageal squamous cell carcinoma tissues and paired normal esophageal tissues (Figures 7–8, Table 3) and analyzed the expression differences. Target genes with significant expression differences were selected for qRT-PCR validation.

SPP1, also known as osteopontin, is an integrin-binding protein encoded on human chromosome 4 (4q13). It is also a secreted glycoprotein that can be secreted from various types of cells, including macrophages, endothelial cells, and osteoclasts. It affects the adhesion, proliferation, differentiation, migration, and survival of tumor cells and has become one of the most popular research genes in recent years. Many studies have found that it is overexpressed in various cancer tissues such as lung cancer, prostate cancer, and hepatocellular carcinoma [66, 67, 94–96]. From the results of the internal reference gene stability verification experiment, the expression level of SPP1 in tumors is increased, but the increase is not uniform, and it also testifies to the speculation that the patient-specific pathology and stage of cancer tissue may lead to different gene expression levels [97]. The data obtained from our final results reveal that the two reference candidate genes, PPIA and RPS18, were the most stably expressed in ESCC. A variety of statistical data supports these results. The higher-ranked reference genes had less variation in expression levels, and higher expression levels (Figure 2), and both genes were ranked high in the different software assessments (Figures 3–6, Tables 1 and 2). For the two commonly used reference genes, ATCB and GAPDH, the ranking was the median in the results of this experiment, indicating that the expression in esophageal squamous cell carcinoma was not stable enough. Similar conclusions have also been drawn from other tumor-related studies [84, 85, 98–100]. In general, internal reference genes should not be used blindly for different experiments. The selection of internal reference genes and numbers should be based on different experimental materials, conditions, and purposes.

## 7. Conclusion

Lack of normalization may lead to an inaccurate conclusion, absolute quantification of reference genes is not recommended. This research systematically explored the applicability of candidate endogenous reference genes for normalization and performed a comprehensive assessment of reference gene required for qRT-PCR analysis of target gene expression in ESCC. It proved that RPS18 and PPIA are reference gene with a relatively stable expression before and after the onset of ESCC. Statistics are reliable. It proposes PPIA or RPA18 or the combination of the two candidate genes as the preferred reference genes when studying the molecular mechanisms of ESCC pathogenesis and gene profiles. While GUSB is the most unstable and is not recommended for normalization, this study confirmed that the expression level of SPP1 is upregulated additionnaly, which has the potential to become a diagnostic and prognostic biomarker for ESCC, combined with and multiscale computational models applied clinically. The amount of SPP1 expressed in different patients most likely related to the patient's clinical features, such as type of differentiation and pathology. In future research, our group would focus on the correlation analysis between the expression level of SPP1 and clinical characteristics to further explore the potential of SPP1 as a biomarker for ESCC.

## Figures and Tables

**Table 1:** Evaluation of the expression stability of the eight candidate internal reference genes by *NormFinder.*

Rank number	Cancer esophageal tissues		Normal esophageal tissues		All esophageal tissues	
	Gene in ranking order	Stability value	Gene in ranking order	Stability value	Gene in ranking order	Stability value
1	PPIA	0.51	RPS18	0.65	GAPDH	0.15
2	RPS18	0.64	PPIA	0.84	PPIA	0.19
3	ACTB	0.77	B2M	1.00	ACTB	0.25
4	PGK1	0.94	HPRT1	1.31	B2M	0.4
5	GAPDH	0.96	GAPDH	1.40	HPRT1	0.56
6	B2M	1.13	ATCB	1.82	RPS18	0.74
7	HPRT1	1.17	GUSB	2.17	GUSB	0.87
8	GUSB	1.54	PGK1	3.23	PGK1	1.03

Note: The lower the SV, the higher the stability.

**Table 2:** Evaluation of the expression stability of the eight candidate internal reference genes by *BestKeeper.*

Group	Stability ranking	Gene name	Std dev [±CP]
Cancer esophageal tissues	1	PPIA	0.98
	2	RPS18	0.99
	3	ATCB	1.00
	4	PGK1	1.04
	5	GUSB	1.09
	6	B2M	1.17
	7	HPRT1	1.29
	8	GAPDH	1.42

Normal esophageal tissues	1	PPIA	0.77
	2	RPS18	0.78
	3	B2M	0.81
	4	HPRT1	1.12
	5	GAPDH	1.37
	6	ACTB	1.58
	7	GUSB	1.77
	8	PGK1	2.55

All esophageal samples	1	RPS18	0.89
	2	PPIA	1.09
	3	B2M	1.37
	4	ATCB	1.38
	5	GUSB	1.44
	6	GAPDH	1.54
	7	HPRT1	1.61
	8	PGK1	2.43

**Table 3:** SPP1 gene expression difference statistics.

SPP1 gene expression statistics						
	E(NO)	E(CA)	log2FC	*P*	Significant	Regulate
NO vs. CA	3.77	79.07	4.34	0	Yes	Up

Note: E represents TPM; FC represents CA is divided by NO.

## Data Availability

The datasets used and/or analyzed during the current study are available from the corresponding author upon reasonable request.

## Supplementary Materials

Supplementary Materials This section includes the extraction and quality verification of RNA templates, the specificity and amplification efficiency of eight internal reference primers, and the methods of five stability evaluation software, and it can be seen in Figure S1 that all templates are of good quality, the primer bands are single, and they have good expression at different concentrations of templates.
