# Supplementary material for: Identification and Validation of Common Reference Genes for Normalization of Esophageal Squamous Cell Carcinoma Gene Expression Profiles
Source: Biomed Res Int. 2022 Nov 23;2022:9125242. doi: 10.1155/2022/9125242 (PMC9711964; doi:10.1155/2022/9125242)
Supplement: Supplementary Materials — This section includes the extraction and quality verification of RNA templates, the specificity and amplification efficiency of eight internal reference primers, and the methods of five stability evaluation software, and it can be seen in Figure S1 that all templates are of good quality, the primer bands are single, and they have good expression at different concentrations of templates. [file 9125242.f1.zip › Supplement (1).docx]

**Material and Methods**

**RNA extraction and cDNA synthesis**

All cancer tissues and normal tissue samples were mixed to form two mixtures for transcriptome sequencing, with three replicates (Majorbio, China). Total RNA was extracted with a Trizol reagent according to the manufacturer's protocol (Invitrogen, USA). Detection of RNA integrity by 1% agarose gel electrophoresis. Measured the concentration and A260/280 ratio of purified RNA with Nanodrop ND-1000 (Thermo Scientific, Wilmington, DE, USA). Aspirating 1μg of total RNA and using a reverse transcription kit for cDNA synthesis and genomic RNA removal according to the manufacturer's protocol (Vazyme Biotech, China)

**Reference gene selection and primer design**

The eight genes initially selected as candidate reference genes included the ACTB, GAPDH, and so on (Table s1). Primers were designed using Primer 5.0 software or refer to other literature. The specificity of each primer pair was evaluated by 1% agarose gel electrophoresis. The PCR amplification efficiency (E) and correlation coefficient (R^2^) for each primer pair were calculated with a tenfold cDNA dilution series.

Table s1 Potential reference genes evaluated in this study

| Gene | Gene name | Gene id | Genomic localization | Description |
| --- | --- | --- | --- | --- |
| ACTB | actin beta | 60 | 7p22.1 | Cytoskeletal structural protein |
| GAPDH | glyceraldehyde-3-phosphate dehydrogenase | 2597 | 12p13.31 | Oxidoreductase in glycolysis  and gluconeogenesis |
| RPS18 | ribosomal protein S18 | 6222 | 6p21.32 | Ribosome subunit |
| B2M | beta-2-microglobulin | 567 | 15q21.1 | The beta chain portion of HLA |
| HPRT1 | Hypoxanthine  phosphoribosyl transferase 1 | 3251 | Xq26.2-q26.3 | Metabolic salvage of purines |
| GUSB | glucuronidase beta | 2990 | 7q11.21 | Hydrolytic degradation of glycosaminoglycans |
| PPIA | peptidylprolyl isomerase A | 5478 | 7p13 | Encode PPIase, accelerating the folding of proteins |
| PGK1 | phosphoglycerate kinase 1 | 5230 | Xq21.1 | catalyzes the conversion of 1,3-diphosphoglycerate to 3-phosphoglycerate |

Note：HLA: human leukocyte antigen; PPIase: the peptidylprolyl cis-trans isomerase(PPIases catalyzes the cis-trans isomerization of proline amide peptide bonds in oligopeptides and accelerates protein folding)

**Method of the five algorithms-specific content**

The comparative *delta-Ct* method: We used the comparative delta-Ct method, which bypasses the potential source of error of poor precision and reproducibility of total RNA normalization, to confidently identify usefulness by comparing the relative expression of "gene pairs" in each sample housekeeping genes^1^.

*GeNorm*: Specifically, the GeNorm algorithm calculates the expression stability value (M) and pairwise variation (V), and the most stably expressed gene is the one with the lowest M value^2,3^. For optimal data normalization, qRT-PCR analysis is generally more stable when two or more reference genes are used. Moreover, the GeNorm algorithm determines the optimal number of reference genes according to the pairwise variation Vn/Vn+1. If Vn/Vn+1 is less than 0.15, n is the most suitable number of internal reference genes. In contrast, if Vn/Vn+1 is greater than 0.15, n + 1 is the ideal number of internal reference gene^4^.

*NormFinde*r: An add-in for Microsoft Excel which adds the NormFinder functionality directly to the Excel software package. It calculates the stability values (SV) for each gene based on the variance analysis, and the gene with the lowest SV is identified as the most stably expressed gene^5^.

*BestKeeper*: An Excel-based tool. It mainly determines the stability of reference gene expression based on SD, r, and CV of the Ct data for all reference genes^6^.

RefFinder: The program *RefFinder* integrates four analysis programs, including *GeNorm*, *NormFinder*, *BestKeeper*, and the *delta Ct* evaluation method^7,8^. The reference genes were ranked based on the geometric mean (GM) values calculated with RefFinder (http://blooge.cn/RefFinder/). It is a software for comprehensive analysis of reference genes.

**Result**

**RNA quality assessment**

We evaluated the quality of the RNA as the starting material (Figure s1). Firstly, the A260/280 ratio was determined by Nanodrop ND-1000, which was 2.03±0.11 (mean± SD) for tumor tissue and 2.04±0.05 for normal tissue, which also confirmed good RNA quality without protein and DNA contamination (Figure s1C). Secondly, performed agarose gel electrophoresis on the paired mixed RNA that was sequenced by the company, found that the bands were normal (Figure s1A), and then used RNA 6000 Nano Labchip for the paired mixed tissues. Finally, the RNA integrity number (RIN) of the normal tissue is 9.63 ± 0.12 (mean ± SD) and 9.53 ± 0.2 (mean ± SD) for patient tissue samples. For paired tissue samples, we did not find statistically significant differences in A260/280 ratios (One-way ANOVA P=0.487) or RIN (Paired Student's t-test, P=0.507) between normal and tumor tissues.


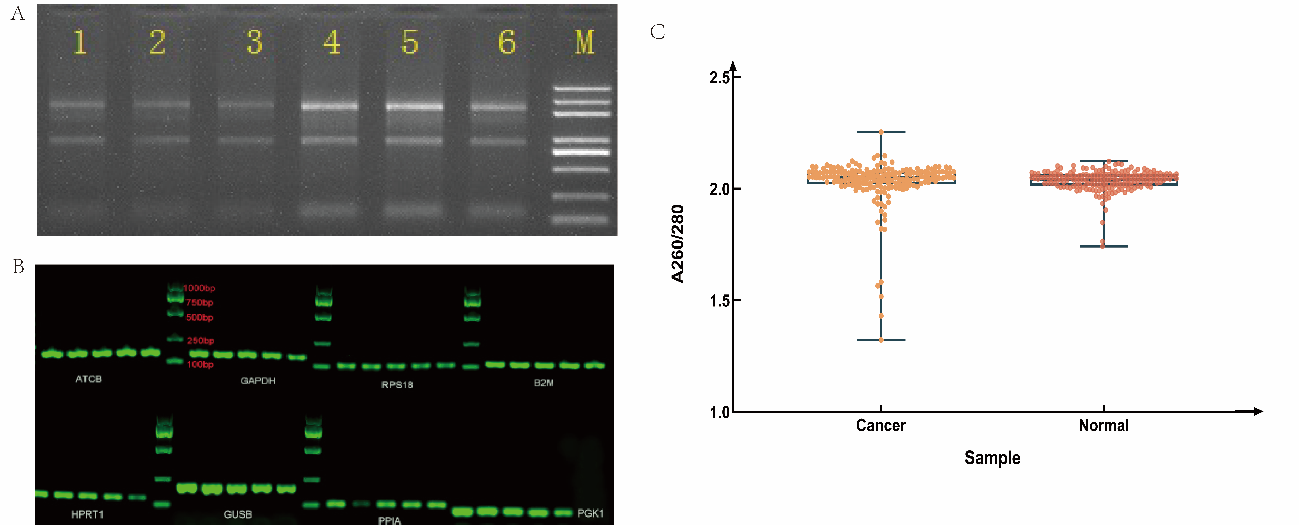


Figure s1 RNA sample and primer quality verification. A, RNA Integrity Verification - Nucleic Acid Gel Image; B, Agarose gel electrophoresis analysis of primer specificity; C, A260/280 distribution of all RNA samples

Note: A, 1-6:1-3, Cancer tissue; 4-6, Normal tissue; M, marker; B, Nucleic acid bands from left to right are 10-fold gradient concentrations, from high to low

**3.3 Specificity of candidate reference genes** **primer**

The primer specificity was determined by gel electrophoresis and melting curve analysis. All primers amplified a single amplicon band of expected size under different concentration gradients (Figure s1B). The Melting curve analysis also displayed only a single peak and determined the amplification efficiency of each primer set (Table s2). The amplification efficiencies of the eight candidate reference genes ranged from 95.8% (RPS18) to 103.2% (GUSB), and the correlation coefficient R2 ranged from 0.9801 (GUSB) to 0.9995 (PGK1). It indicates that the primers designed by the selected genes have high specificity and similar good amplification efficiency, which can be suitable for subsequent qRT-PCR analysis.

Table s2 Primers and PCR amplification efficiency of candidate reference gene

| Gene | Primer sequence | Produce size | R^2^ | slope | E (100%) |
| --- | --- | --- | --- | --- | --- |
| ACTB | F: CCTGGCACCCAGCACAAT  R: GGGCCGGACTCGTCATAC | 144 | 0.9985 | -3.3112 | 100.4497 |
| GAPDH | F: CAGGAGGCATTGCTGATGAT  R: GAAGGCTGGGGCTCATTT | 138 | 0.9801 | -3.4169 | 96.1837 |
| RPS18 | F: ATCCTCAGTGAGTTCTCCCG  R: CTTTGCCATCACTGCCATTA | 106 | 0.9983 | -3.4251 | 95.8675 |
| B2M | F: ACTGAATTCACCCCCACTGA  R: CCTCCATGATGCTGCTTACA | 114 | 0.9922 | -3.2568 | 102.7916 |
| HPRT1 | F: CCTGGCGTCGTGATTAGTGAT  R: AGACGTTCAGTCCTGTCCATAA | 131 | 0.9979 | -3.4035 | 96.7050 |
| GUSB | F: GGTCCTAGGCTCCGTATGTG  R: GCTCAACACTGCTTACCTGG | 201 | 0.9962 | -3.2465 | 103.2469 |
| PPIA | F: GTCAACCCCACCGTGTTCTT  R: CTGCTGTCTTTGGGACCTTGT | 97 | 0.9918 | -3.3796 | 97.6482 |
| PGK1 | F： GCTGCTGGGTCTGTCATCCT  R: TCTTTTCCCTTCCCTTCTTCCT | 68 | 0.9995 | -3.3636 | 98.2898 |

Note: High or low amplification efficiency will affect the accuracy of experimental data. Theoretically, the optimal value is 100%, indicating that the template is copied exponentially, and it is controlled between 90% and 110%, which is the accepted error range nowadays ^9^.

Table s3 Glossary of Abbreviations

| Full name | Abbreviation |
| --- | --- |
| Esophageal squamous cell carcinoma | ESCC |
| Quantitative Real-Time Polymerase Chain | qRT-PCR |
| Esophageal adenocarcinoma | EAC |
| *actin* beta | ATCB |
| glyceraldehyde-3-phosphate dehydrogenase | GADPH |
| Ribosomal protein S18 | RPS18 |
| beta-2-microglobulin | B2M |
| Hypoxanthine phosphoribosyl transferase 1 | HPRT1 |
| glucuronidase beta | GUSB |
| peptidylprolyl isomerase A | PPIA |
| phosphoglycerate kinase 1 | PGK1 |
| human leukocyte antigen | HLA |
| the peptidyl-prolyl cis-trans isomerase | PPIase |
| Minimum Information for Publication of Quantitative Real-Time PCR Experiments | MIQE |
| Transcripts Per Million reads | TPM |
| stability values | SV |
| Normal esophageal tissues | NO |
| Cancer esophageal tissues | CA |
| All esophageal tissues | ALL |
| standard deviation | SD |
| Pearson correlation coefficient | r |
| the coefficient of variation | CV |
| The fold difference | FC |
| protein-protein interaction | PPI |
| secreted phosphor protein 1 | SPP1 |
| relative expression | RE |

1 Gong, Z. K. *et al.* Identification and validation of suitable reference genes for RT-qPCR analysis in mouse testis development. *Molecular Genetics & Genomics* **289**, 1157-1169 (2014).

2 Goto, M. *et al.* CXCR4 Expression is Associated with Poor Prognosis in Patients with Esophageal Squamous Cell Carcinoma. *Annals of Surgical Oncology* (2015).

3 A. *et al.* Utility of the Housekeeping Genes 18S rRNA, β-Actin and Glyceraldehyde-3-Phosphate-Dehydrogenase for Normalization in Real-Time Quantitative Reverse Transcriptase-Polymerase Chain Reaction Analysis of Gene Expression in Human T Lymphocytes. *Scandinavian Journal of Immunology* (2004).

4 Pisamai, S., Rungsipipat, A., Kalpravidh, C. & Suriyaphol, G. Gene expression profiles of cell adhesion molecules, matrix metalloproteinases and their tissue inhibitors in canine oral tumors. *Research in Veterinary Science* **113**, 94-100 (2017).

5 Hyrskyluoto, A. Detection of Hypoxia-inducible mRNAs in the Plasma of Non- Small Cell Lung Cancer Patients. (2009).

6 Sedano, M. J., Ramos, E. I., Choudhari, R., Harrison, A. L. & Gadad, S. S. Hypoxanthine Phosphoribosyl Transferase 1 Is Upregulated, Predicts Clinical Outcome and Controls Gene Expression in Breast Cancer. *Cancers* **12** (2020).

7 Magdalena *et al.* Identification of suitable reference genes for gene expression measurement in uterine sarcoma and carcinosarcoma tumors. *Clinical Biochemistry* **45**, 368-371 (2012).

8 Vagnoni, V. *et al.* Molecular diagnostic tools for the detection of nodal micrometastases in prostate cancer patients undergoing radical prostatectomy with extended pelvic lymph node dissection: a prospective study. *Urologia*, 0-0 (2012).

9 Tiziana *et al.* Selection of reference genes for diurnal and developmental time-course real-time PCR expression analyses in lettuce. *Plant Methods* (2016).
