# Supplementary figures and images for: Identification and Validation of Common Reference Genes for Normalization of Esophageal Squamous Cell Carcinoma Gene Expression Profiles
Source: Biomed Res Int. 2022 Nov 23;2022:9125242. doi: 10.1155/2022/9125242 (PMC9711964; doi:10.1155/2022/9125242)

A

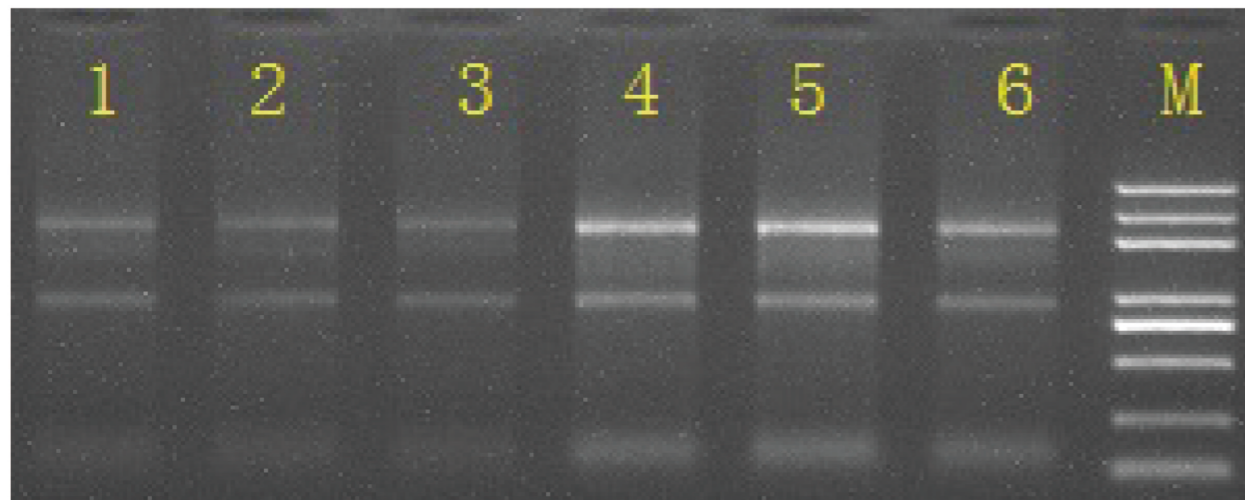

B

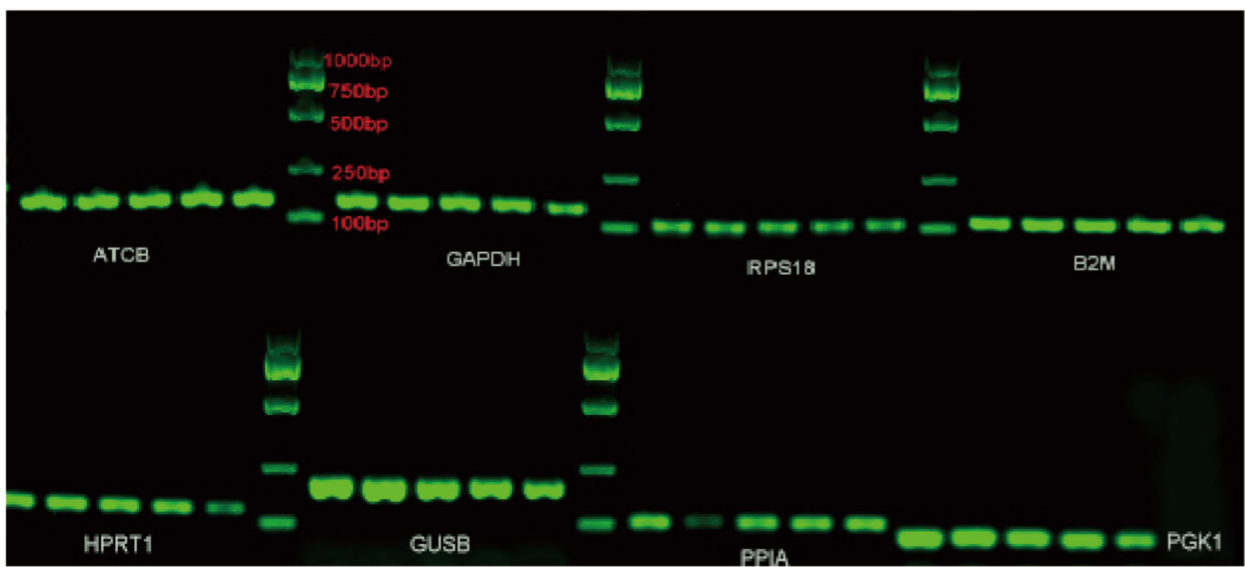

C

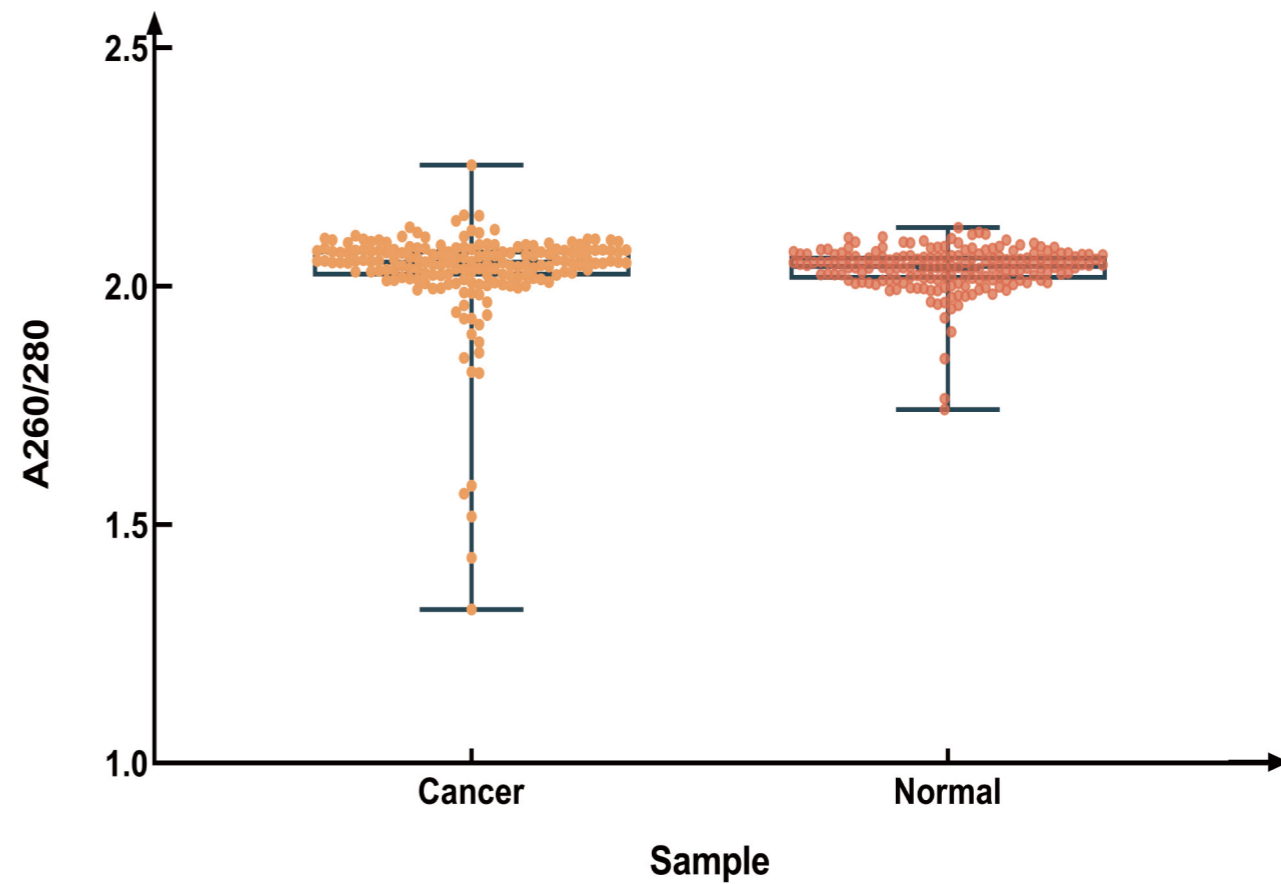

Supplement: Supplementary Materials — This section includes the extraction and quality verification of RNA templates, the specificity and amplification efficiency of eight internal reference primers, and the methods of five stability evaluation software, and it can be seen in Figure S1 that all templates are of good quality, the primer bands are single, and they have good expression at different concentrations of templates. [file 9125242.f1.zip › Figures1.pdf]
